# Clinical characteristics of COVID-19 patients treated in emergency COVID-19 hospitals in Vietnam: Experience from Phutho province, Vietnam

**DOI:** 10.7150/ijms.94461

**Published:** 2024-04-22

**Authors:** Ngoc Huy Nguyen, Khanh Minh Tran, Lee Hoon Jong, Son Thanh Dinh Le, An Quang Nguyen, Duc Minh Duong, To Dang Nguyen, Lan Ngoc Thi Nguyen, Lan Thi Phuong Dam, Tuan Ngoc Minh Nguyen

**Affiliations:** 1 Phutho Department of Health, Phutho province, Vietnam.; 2 Phutho General Hospital, Phutho province, Vietnam.; 3 Science & Research Center, Seoul National University College of Medicine, Seoul, Seoul Metro, Republic of Korea.; 4 Public Health Center Director, Geoje Public Health Center, Geoje city, Gyeongsangnam-do, Republic of Korea.; 5 Hanoi University of Public Health, Vietnam.; 6 Clinical Laboratory, Hanoi Medical University Hospital, Vietnam.; 7 Biochemistry Department, Hanoi Medical University, Vietnam.; 8 Phutho Medical Colleage, Phutho province, Vietnam.; 9 Vietnam National University-University of Medicine and Pharmacy, Hanoi, Vietnam

**Keywords:** COVID-19, vaccination, clinical characteristics, diagnosis and treatment, Vietnam

## Abstract

**Background:** This study aimed to evaluate the clinical characteristics, patient's management approaches, and outcomes of the COVID-19 patients in Phu Tho Province, Vietnam.

**Methods:** A retrospective, multicenter study of 2166 COVID-19 patients in 13 hospitals in Phutho Province, Vietnam. The subjects were divided into 3 groups based on vaccination status: unvaccinated group, 1^st^ dose of vaccine group, 2^nd^ dose of vaccine group. The clinical characteristics, management approaches, and outcomes were collected and compared between the 3 groups.

**Results:** The hospitalization rate of the 3 groups decreased from the unvaccinated group, the 1^st^ dose of vaccinated group, to the 2^nd^ dose of vaccinated group, 42.61%; 30,24% and 27,15% respectively. The 19-40 years old group had the highest hospitalization rate (38,1%) together with the group that had not accepted the full COVID 19 vaccination dose (57,64%). The 2^nd^ dose of vaccinated group had the lowest percentages of high temperature, cough, dyspnea, chest pain and sore throat. The unvaccinated group had the highest heart rate, respiratory rate and SpO2 compared to the two other groups. The percentage needing Immunomodulation and Anticoagulant Therapy was highest (6.8% and 1.4 % respectively) in the unvaccinated group. The percentage receiving Antiviral Therapy was highest (42,5%) in those who had received the 2^nd^ dose of vaccine.

**Conclusions:** COVID-19 vaccination improved the symptoms of the patients and should be accepted in all ages.

## Introduction

The COVID-19 pandemic originated from Wuhan, China at the end of 2019 and has had a significant impact on public health systems and economies worldwide 1, 2. As the closest neighboring country to China, Vietnam was affected as soon in January 2020 3. Experiencing four waves of COVID-19, Vietnam has reported over 200,000 confirmed cases and more than 4,000 deaths due to COVID-19, reflecting the damage of COVID-19 disease in the country 4.

Phutho is a province located in the North-Eastern region of Vietnam and was severely affected by the pandemic. Despite proactively preventing COVID-19 disease by various solutions such as 5K, vaccination, telemedicine and government policies 3, there were over 5,500 confirmed cases and 10 deaths reported during the period of COVID-19 in Phutho province. Aware that this emerging infectious disease was serious and there was a need to understand the features of the disease and impact on patients and staff we implemented a system to monitor the clinical characteristics of the patients. Therefore, this study has been conducted aiming to present a comprehensive evaluation of the clinical characteristics, management approaches, and outcomes of the COVID-19 patients based on our experience and medical records of COVID-19 patients in Phutho Province, Vietnam.

## Methods

### Study design and setting

This study was a retrospective, multicenter study.

### Study population

A total of 2166 COVID-19 patients from 13 hospitals in Phutho province were identified and were divided into 3 groups based on vaccination status: unvaccinated group, 1^st^ dose of vaccination group, 2^nd^ dose of vaccination group.

The inclusion criteria were: All admitted patients caused by COVID-19 between 1^st^ October 2021 and 31^st^ December 2021; diagnosed and confirmed as SARS-CoV-2 positive by polymerase chain reaction (PCR) from a nasopharyngeal swab.

### Data collection

The demographic features collected were age, gender, medical history, comorbidities, the patient's clinical characteristics (symptoms, vital signs, pulse oxygen saturation (SPO_2_, %) at rest and exertion), the laboratory data including white blood cell (WBC), hemoglobin (HGB), hematocrit, and the therapy were collected from the electronic medical records.

### Data statistical analysis

Categorical variables were presented as numbers (percentage) and compared using the chi-square test or Fisher's exact test; while continuous variables were presented as medians (interquartile ranges) and compared using the Kruskal-Wallis test. Statistical significance was confirmed if *p*-value was <0.05. Statistical analyses and figure were performed by using the IBM SPSS Statistics software 26.0 (SPSS, Inc., Chicago, IL).

## Results

### Subject's characteristics

Subject's characteristics are provided in Table 1. A total of 2166 COVID-19 cases were enrolled in our analysis (51.6% female and 48.4% male). 923 cases were unvaccinated, while 655 and 588 cases were vaccinated with 1^st^ and 2^nd^ dose, respectively. The majority of patients were self-employed (1048) with 192 employed (*p*<0.001). Similarity, the Kinh population (as the majority) (1193) and minority (173) were different significantly between groups (*p*<0.001). The median age of the patients was 30.0 (IQR 14-41.2). The vaccinated cohort was significantly older than the unvaccinated cohort (median [IQR 25-75%]: 36 [27-46] and 36.5 [30-47.4] vs 13.0 [9-26], *p*<0.001), with comorbidities significantly more frequent in the vaccinated groups: hypertension, diabetes mellitus, COPD, taking corticosteroids, smoking and alcohol were all significant at *p*<0.05. Clinical presentations were significantly presented in the unvaccinated group with 548 cases versus 419 (1^st^ dose) and 330 cases (2^nd^ dose), respectively.

Table 2 showed the clinical symptoms of the subjects at the time of hospital admission. Significant differences were observed between vaccinated and unvaccinated patients, in the prevalence of elevated body temperature ≥37.5°C, cough, dyspnea, fatigue, headache, chest pain, gustatory dysfunction, olfactory dysfunction, and myalgia (p<0.05).

Body temperature ≥37.5°C showed a decreasing trend among vaccinated individuals, with a prevalence of 20.5% among unvaccinated individuals, 18.3% among first dose recipients, and 13.4% among second dose recipients (p = 0.002). Cough demonstrated increasing prevalence rates among vaccinated individuals, with a prevalence of 50.9% among unvaccinated individuals, 56.8% among first dose recipients, and 46.6% among second dose recipients (p = 0.001). Dyspnea exhibited decreasing prevalence rates among vaccinated individuals, with a prevalence of 20.6% among unvaccinated individuals, 16.3% among first dose recipients, and 8.8% among second dose recipients (p < 0.001). Fatigue, headache, and chest pain showed a similar pattern, with higher prevalence rates among first dose recipients and slightly lower rates among second dose recipients compared to unvaccinated individuals (p < 0.001). Gustatory dysfunction demonstrated increasing prevalence rates among vaccinated individuals, with a prevalence of 5.0% among unvaccinated individuals, 9.3% among first dose recipients, and 10.4% among second dose recipients (p < 0.001). Olfactory dysfunction displayed a similar trend, with prevalence rates of 3.7% among unvaccinated individuals, 6.1% among first dose recipients, and 8.0% among second dose recipients (p = 0.001). Myalgia also showed significant differences, with prevalence rates of 3.1% among unvaccinated individuals, 6.3% among first dose recipients, and 5.1% among second dose recipients (p = 0.012).

There were no significant differences in the prevalence of these symptoms within three groups (p > 0.05), including feeling cold, diarrhea, rhinorrhea (runny nose), nausea/vomiting.

Table 3 showed the clinical examination characteristics; the laboratory results and the treatment given for the three groups.

Significant differences were found in maximum blood pressure, minimum blood pressure, heart rate, respiratory rate, and oxygen saturation (SpO2) between the vaccination groups. The results of laboratory parameters presented significant differences in white blood cell counts, hemoglobin levels, and hematocrit percentages between the vaccination groups (p<0.05). The white blood cell of the unvaccinated group, the 1st dose vaccination recipients, and the 2^nd^ dose vaccination recipients were 6.86 ± 5.75 g/L, 6.92 ± 4.95 g/L and 7.85 ± 2.84 g/L respectively (p = 0.031). The hemoglobin of the unvaccinated group, the 1st dose vaccination recipients, and the 2^nd^ dose vaccination recipients were 136.21 ± 28.24 g/L, 139.64 ± 24.72 g/L and 141.48 ± 22.11 g/L respectively (p = 0.004). The hematocrit of the unvaccinated group, the 1st dose vaccination recipients, and the 2^nd^ dose vaccination recipients were 41.56 ± 5.26, 42.69 ± 5.79, and 42.96 ± 5.30 respectively (p < 0.001) (Table 3).

The percentage of patients given Molnupiravir Therapy was highest in the 2^nd^ dose vaccination recipient group. The percentages of subjects who received Molnupiravir in the unvaccinated group, the 1st dose vaccination recipients, and the 2^nd^ dose vaccination recipients were 2.7%; 6.6%; 40,8% respectively. The percentage of patients using Dexamathasone Therapy and or Levanox Therapy were lowest in the 2^nd^ dose vaccination recipient group. The percentages of subjects who received Dexamethasone of the unvaccinated group, the 1st dose vaccination recipients, and the 2^nd^ dose vaccination recipients were 4.2%; 6.1%; 2.7% respectively (p= 0.014). The percentages of subjects who received Levanox of the unvaccinated group, the 1st dose vaccination recipients, and the 2^nd^ dose vaccination recipients were 1.4%; 3.2%; 1.2% respectively (p = 0.012). There were no significant differences in the rate of using Remdesivir, Heparin, Methyl in three groups (Table 3).

## Discussion

This was an epidemiological study of COVID-19 hospitalized patients in Phu Tho province of Vietnam. The study focused on differences in age, gender, ethnicity, occupations, clinical presentation and comorbidities between patients who were unvaccinated compared to those who had received either one or two doses of the vaccine.

As a result of government policy, the number of vaccinated people was higher than the number of unvaccinated people. According to vaccine strategy, the Vietnam Ministry of Health prioritized older people who may be more vulnerable to severe disease outcomes to receive the vaccine. This strategy was similar in some other countries during the pandemic limitation of vaccines 5.

Age is a well-established risk factor for severe COVID-19 outcomes, with older individuals being more susceptible to severe illness and complications 6. In this study, the median age of the study population was 30.0 years (interquartile range [IQR]: 14-41.2). Notably, the unvaccinated cohort were mainly under 18 years old, while the vaccinated groups were over 19 years old, which resulted in a significantly higher median age compared to the unvaccinated cohort (36 [IQR: 27-46] and 36.5 [IQR: 30-47.4] vs. 13.0 [IQR: 9-26], p<0.001). The results were similar to other reported studies which showed that most COVID-19 infections occurred in the 19-30-year age groups 7, 8. The high frequency of COVID-19 infection in these age groups may be due to inclusion of mainly working-age population in this age group 9. In this study, the proportion of female patients (51.6%) was slightly higher than male patients (48.4%). These findings were consistent with previous studies reporting a similar gender distribution in COVID-19 cases 7, 10-12.

Comorbidities have been identified as significant risk factors for severe COVID-19. In this study the proportion of individuals with comorbidities was small, possibly due to the young age group of patients. The most frequent comorbidities were hypertension, diabetes, chronic obstructive pulmonary disease (COPD), corticosteroid use, smoking, and alcohol consumption in the vaccinated cohorts (p<0.05). It seems that the frequency of COVID-19 patients with comorbidities was hypertension and cardiovascular association 8. Our data has not covered the effect of vaccination on occurrence of long-term sequelae and this warrants further research in Vietnam. A previous study has suggested that vaccinated patients who then had COVID-19 had a worse quality of life six months after admission with the infection than unvaccinated patients, although this could have been explained by the higher mean age and disease burden in the vaccinated group 31. Additionally, the results of our data on clinical presentation showed that the majority of patients presented COVID-19 features rather than being asymptomatic.

Clinical presentations provide valuable insights into the manifestations and severity of COVID-19. The significant clinical presentations of COVID-19 patients in this study were cough, followed by sore throat and fatigue, which were seen in previous studies 7. The chest pain symptoms of COVID-19 were also reported as a common manifestation as other studies 13-16. Indeed, our results showed that the unvaccinated group exhibited a significantly higher number of clinical presentations than the vaccinated group, which also agree with results of previously published studies. Notably, body temperature of COVID-19 patients ≥ 37.5°C among vaccinated individuals displayed lower prevalence rates compared to unvaccinated individuals. This result might be the reason that many medical facilities used temperature screening as an appropriate indicator for monitoring patients during the pandemic 17, 18. Cough, sore throat, fatigue and chest pain were common symptoms of COVID-19 19, 20 which made it difficult to differentiate between COVID-19 and other respiratory diseases 21, 22. Some patients in this study had symptoms of diarrhea, nausea and vomiting suggesting the possible involvement of the gastrointestinal tract 23. Other individuals had cold feeling and dysfunction of gustation or olfaction which were possibly temporary side effects of the immune response to vaccination 24, 25. Studies have shown that using artificial intelligence to interpret radiological findings provides high sensitivity and specificity for the diagnosis of COVID-19 as well as predicting severity 26, 27. Although currently, COVID-19 infection is no longer as serious as in previous periods the disease still appears seasonally in Vietnam as well as many other countries around the world. Along with forms of seasonal flu, covid-19 can also co-infect and aggravate other disease. In addition, in some subjects, symptoms of 'Long COVID' appear, although it can be difficult to determine whether the symptoms are due to COVID 19. These symptoms include fatigue, cough, headache, pain and fatigue in the joints. The results in this study has shown that there is a reduction in these symptoms if patients are vaccinated against COVID-19. Currently, many organizations such as the US CDC still recommend the use of COVID-19 vaccine to prevent disease as well as alleviate long-term or post-COVID-19 symptoms, especially in adults.

Laboratory findings play a crucial role in understanding the pathophysiology and severity of COVID-19. The results revealed significant differences in white blood cell counts, hemoglobin levels, and hematocrit percentages between the vaccinated groups (p<0.05). Unvaccinated individuals had a slightly lower mean white blood cell count (6.86 ± 5.75 g/L) compared to those who received the first dose (6.92 ± 4.95 g/L), while participants who received the second dose had the highest mean count (7.85 ± 2.84 g/L, p = 0.031). Regarding hemoglobin levels, unvaccinated individuals had a slightly lower mean (136.21 ± 28.24 g/L), whereas individuals who received the first dose had a slightly higher mean (139.64 ± 24.72 g/L), and those who received the second dose had the highest mean hemoglobin level (141.48 ± 22.11 g/L, p = 0.004). Similarly, significant variations were observed in hematocrit percentages, with higher means observed among the vaccinated groups compared to the unvaccinated group (41.56 ± 5.26, 42.69 ± 5.79, and 42.96 ± 5.30, respectively, p < 0.001). These changes in COVID-19 showed the ability of adaption to the COVID-19 infection. Many researchers have demonstrated that during the Covid-19 infection, the red blood cell count changed to adapt to the hypoxia. The unvaccinated group had the lower SpO2 index that could increase the hemoglobin and hematocrit index. The slight increase of white blood cells has been reported in many studies. The white blood cell count in the day of hospital admission is associated with the immune capability of the body. These findings suggest that vaccination status may have an impact on hematological parameters, potentially reflecting differences in disease severity or immune response.

The length of hospitalization and vital signs were also assessed in relation to vaccination status.

Vietnam is statistically one of the countries with a high COVID-19 vaccination rate, with the announced rate up to 97%. However, this study also shows that a factor that greatly affects acceptance or willingness to get vaccinated against COVID-19 is occupation. In this study, there was a significantly higher number of self-employed and workers belonging to unvaccinated group, and infected COVID-19 compared to other occupations. Other studies showed that healthcare staff had higher potential for getting the disease than the others because of exposure to infection in the hospital environment 28, 29. Further investigations should be conducted to explore the specific factors contributing to the increased risk among these occupational groups 30.

The study had some limitations. The first limitation was that not all required data were available from the electronic record. So that it was difficult to estimate exactly the pathological changes in all the subjects. The second limitation was the study enrolled all the hospitalized COVID-19 subjects, so that the high percentage of the subjects in unvaccinated group were under 18. Because at that time, using vaccines for young children was not widely accepted by parents and they were not a priority group for government policy on vaccination. That made it difficult when comparing the rate of the symptoms in all the groups. Besides, it is essential to evaluate other factors that affected to the results, such as the specific vaccine types used, the timing of symptom onset in relation to vaccination, and individual variations. Further investigations should consider these factors to establish a causal relationship between vaccination and symptomatology.

Overall, the findings of this study provide important insights into the laboratory findings, treatment patterns, and vital signs among COVID-19 patients in relation to their vaccination status. The observed differences in laboratory parameters, treatment utilization, and vital signs highlight the need for further investigation to better understand the impact of vaccination on disease outcomes and guide clinical management strategies.

## Conclusion

Covid 19 vaccination could improve the symptoms of the patients and should be accepted in all ages.

## Figures and Tables

**Table 1:** General and clinical characteristics of COVID-19 admitted patients in multicentral in Phutho, Vietnam from 1^st^ October 2021 to 31^st^ December 2021.

Variables	Total (N=2166)	Unvaccinated (N= 923, 42.61%)	1^st^ dose Vaccinated (N=655, 30.24%)	2^nd^ dose Vaccinated (N=588, 27.15%)	*p* value
Age (median [IQR 25-75%])	30.0 (14-41.2)	13.0 (9-26)	36 (27-46)	36.5 (30-47.4)	
**Age category (n, %)**					**<0.001**
≤ 5 years old	105 (4.8)	96 (10.4)	6 (0.9)	3 (0.5)	
6-18 years old	660 (30.5)	559 (60.6)	88 (13.4)	13 (2.2)	
19-40 years old	826 (38.1)	151 (16.4)	325 (49.6)	350 (59.5)	
41-60 years old	391 (18.1)	66 (7.2)	161 (24.6)	164 (27.9)	
≥ 61 years old	184 (8.5)	51 (5.5)	75 (11.5)	58 (9.9)	
Gender					0.363
Female	1118 (51.6)	461 (49.9)	342 (52.2)	315 (53.6)	
Male	1048 (48.4)	462 (50.1)	313 (47.8)	273 (46.4)	
**Ethnicity**					**<0.001**
Majority (Kinh)	1193 (92.0)	880 (95.3)	599 (91.5)	514 (87.4)	
Minority (Other)	173 (8.0)	43 (4.7)	56 (8.5)	74 (12.6)	
**Occupations**					**<0.001**
Children (define)	161 (7.4)	147 (15.9)	7 (1.1)	7 (1.2)	
Pupils, students	634 (29.3)	515 (55.8)	95 (14.5)	24 (4.1)	
Officials	54 (2.5)	4 (0.4)	18 (2.7)	32 (5.4)	
Workers	192 (8.9)	20 (2.2)	107 (16.3)	65 (11.1)	
Self-emplyed	1048 (48.4)	215 (23.3)	401 (61.2)	432 (73.5)	
Retired	77 (3.6)	22 (2.4)	27 (4.1)	28 (4.8)	
**Clinical presentations**					**0.017**
None	869 (40.1)	375 (40.6)	236 (36.0)	258 (43.9)	
Yes	1297 (59.9)	548 (59.4)	419 (64.0)	330 (56.1)	
**Comorbidities**					
Hypertension	56 (2.6)	13 (1.4)	15 (2.3)	28 (4.8)	**<0.001**
Cardiovascular disease	8 (0.4)	3 (0.3)	1 (0.2)	4 (0.7)	0.297
Diabetes mellitus	35 (1.6)	7 (0.8)	10 (1.5)	18 (3.1)	**0.002**
COPD	18 (0.8)	2 (0.2)	7 (1.1)	9 (1.5)	**0.017**
Kidney disease	1 (0.0)	1 (0.1)	0 (0)	0 (0)	0.510
Corticosteroid use	6 (0.3)	0 (0)	1 (0.2)	5 (0.9)	**0.007**
Stroke patients associated with COVID-19	3 (0.1)	3 (0.3)	0 (0)	0 (0)	0.132
Smoking/Alcohol	39 (1.8)	9 (1,0)	12 (1,8)	18 (3.1)	**0.012**

**Table 2:** Clinical characteristics.

Symptoms	Unvaccinated (923)	1^st^ dose vaccinated (655)	2^nd^ dose vaccinated (588)	*p* value
Body temperature, ≥ 37.5^0^C	189 (20.5)	120 (18.3)	79 (13.4)	**0.002**
Cough	470 (50.9)	372 (56.8)	274 (46.6)	**0.001**
Dyspnea	190 (20.6)	107 (16.3)	52 (8.8)	**<0.001**
Feeling cold	12 (1.3)	20 (3.1)	16 (2.7)	0.041
Fatigue	171 (18.5)	205 (31.3)	164 (27.9)	**<0.001**
Diarrhea	10 (1.1)	15 (2.3)	7 (1.2)	0.117
Headache	37 (4.0)	62 (9.5)	62 (10.5)	**<0.001**
Chest pain	191 (20.7)	96 (14.7)	68 (10.5)	**<0.001**
Gustatory dysfunction	46 (5.0)	61 (9.3)	61 (10.4)	**<0.001**
Olfactory dysfunction	34 (3.7)	40 (6.1)	47 (8.0)	**0.001**
Myalgia	29 (3.1)	41 (6.3)	30 (5.1)	**0.012**
Rhinorrhea	99 (10.7)	69 (10.5)	66 (11.2)	0.922
Sore throat	257 (27.8)	194 (29.6)	117 (19.9)	**<0.001**
Nausea/vomiting	5 (0.5)	2 (0.2)	3 (0.5)	0.776

**Table 3:** Laboratory characteristics and treatment administered

Symptoms	Unvaccinated	1^st^ dose vaccinated	2^nd^ dose vaccinated	*p* value
**Laboratory findings (available, %)**	**667 (44,03%)**	**489 (32,27%)**	**359 (23,70%)**	
White Blood Cells (g/L)	6.86 ± 5.75	6.92 ± 4.95	7.85 ± 2.84	**0.031**
Hemoglobin (g/L)	136.21± 28.24	139.64 ± 24.72	141.48 ± 22.11	**0.004**
Hematocrit (%)	41.56 ± 5.26	42.69 ± 5.79	42.96 ± 5.30	**<0.001**
**Treatments given (n; %)**				
Remdesivir (54; 2.5)	23 (2.5)	21 (3.2)	10 (1.7)	0.236
Molnupiravir (308; 14.2)	25 (2.7)	43 (6.6)	240 (40.8)	**<0.001**
Levanox (41; 1.9)	13 (1.4)	21 (3.2)	7 (1.2)	**0.012**
Heparin (3; 0.1)	1 (0.1)	0 (0)	2 (0.3)	0.260
Dexamethasone (95; 4.4)	39 (4.2)	40 (6.1)	16 (2.7)	**0.014**
Methylprednisolone (54; 2.5)	22 (2.4)	19 (2.9)	13 (2.2)	0.710
**Discharge**				
Length of stay in hospital (days)	> 21 days	15-21 days	8-14 days	**0.001**
Blood pressure max	112.35 ± 36.8	115.97 ± 6.79	116.84 ± 7.58	**0.001**
Diastolic pressure	68.89 ± 9.52	73.23 ± 9.37	74.38 ± 28.08	**<0.001**
Heart rate	88.76 ± 11.65	80.70 ± 6.91	80.02 ± 6.38	**<0.001**
Respiratory rate	21.19 ± 2.61	19.51 ± 1.47	19.50 ± 1.16	**<0.001**
Temperature	36.8 ± 0.22	36.9 ± 1.35	36.85 ± 0.18	0.269
SpO2	98.48 ± 0.78	98.58 ± 0.67	98.62 ± 0.65	**0.001**

## References

[1] Zhang Q, Zhu J, Jia C, Xu S, Jiang T, Wang S (2021). Epidemiology and Clinical Outcomes of COVID-19 Patients in Northwestern China Who Had a History of Exposure in Wuhan City: Departure Time-Originated Pinpoint Surveillance. Front Med (Lausanne).

[2] Geidelberg L, Boyd O, Jorgensen D, Siveroni I, Nascimento FF, Johnson R (2021). Genomic epidemiology of a densely sampled COVID-19 outbreak in China. Virus Evol.

[3] Nguyen NH, Van Nguyen T, Nguyen AQ, Van Nguyen P, Nguyen TNM (2020). The first cohort of the COVID-19 patients in Vietnam and the national response to the pandemic. Int J Med Sci.

[4] Wikipedia COVID-19 pandemic in Vietnam. https://en.wikipedia.org/wiki/COVID-19_pandemic_in_Vietnam.

[5] French J, Deshpande S, Evans W, Obregon R (2020). Key Guidelines in Developing a Pre-Emptive COVID-19 Vaccination Uptake Promotion Strategy. Int J Environ Res Public Health.

[6] Farshbafnadi M, Kamali Zonouzi S, Sabahi M, Dolatshahi M, Aarabi MH (2021). Aging & COVID-19 susceptibility, disease severity, and clinical outcomes: The role of entangled risk factors. Exp Gerontol.

[7] Eid RA, Attia AM, Hassan M, Shaker MA, Kamal MA (2021). Demographic, clinical, and laboratory characteristics of patients with COVID-19 during the second and third waves of the pandemic in Egypt. J Infect Public Health.

[8] Goshayeshi L, Akbari Rad M, Bergquist R, Allahyari A, Hashemzadeh K, Team MC-R (2021). Demographic and clinical characteristics of severe Covid-19 infections: a cross-sectional study from Mashhad University of Medical Sciences, Iran. BMC Infect Dis.

[9] Emami A, Javanmardi F, Pirbonyeh N, Akbari A (2020). Prevalence of underlying diseases in hospitalized patients with COVID-19: a systematic review and meta-analysis. Archives of academic emergency medicine.

[10] Qi X, Liu Y, Wang J, Fallowfield JA, Wang J, Li X (2021). Clinical course and risk factors for mortality of COVID-19 patients with pre-existing cirrhosis: a multicentre cohort study. Gut.

[11] Atalla E, Zhang R, Shehadeh F, Mylona EK, Tsikala-Vafea M, Kalagara S (2020). Clinical Presentation, Course, and Risk Factors Associated with Mortality in a Severe Outbreak of COVID-19 in Rhode Island, USA, April-June 2020. Pathogens.

[12] Sanchez-Pina JM, Rodriguez Rodriguez M, Castro Quismondo N, Gil Manso R, Colmenares R, Gil Alos D (2020). Clinical course and risk factors for mortality from COVID-19 in patients with haematological malignancies. Eur J Haematol.

[13] Sinkeldam M, Buenen AG, Celiker E, van Diepen M, de Vos AM (2022). Characteristics of chest pain in COVID-19 patients in the emergency department. Neth Heart J.

[14] Takase B, Hayashi K, Hisada T, Tsuchiya T, Masaki N, Nagata M (2022). Chest Pain with New Abnormal Electrocardiogram Development after Injection of COVID-19 Vaccine Manufactured by Moderna. Intern Med.

[15] Cunha G, Cerci RJ, Silvestre OM, Cavalcanti AM, Nadruz W, Fernandes-Silva MM (2022). Sex- and Age-Related Impact of the COVID-19 Pandemic on Emergency Department Visits for Chest Pain in Curitiba, Brazil. J Emerg Med.

[16] Aung S, Vittinghoff E, Nah G, Lin A, Joyce S, Mann NC (2021). Emergency activations for chest pain and ventricular arrhythmias related to regional COVID-19 across the US. Sci Rep.

[17] Rasmussen SA, Jamieson DJ (2020). Coronavirus Disease 2019 (COVID-19) and Pregnancy: Responding to a Rapidly Evolving Situation. Obstet Gynecol.

[18] Wang Q, Wang X, Lin H (2020). The role of triage in the prevention and control of COVID-19. Infect Control Hosp Epidemiol.

[19] Chen L, Jin Q, Zhou Y, Yang J, Wang Z, Ge K (2020). Clinical characteristics of 2019 novel coronavirus pneumonia in Zhejiang province, China. Mol Med Rep.

[20] Yang X, Yu Y, Xu J, Shu H, Xia J, Liu H (2020). Clinical course and outcomes of critically ill patients with SARS-CoV-2 pneumonia in Wuhan, China: a single-centered, retrospective, observational study. Lancet Respir Med.

[21] Jiang F, Deng L, Zhang L, Cai Y, Cheung CW, Xia Z (2020). Review of the Clinical Characteristics of Coronavirus Disease 2019 (COVID-19). J Gen Intern Med.

[22] Sun P, Lu X, Xu C, Sun W, Pan B (2020). Understanding of COVID-19 based on current evidence. J Med Virol.

[23] Mohamadian M, Chiti H, Shoghli A, Biglari S, Parsamanesh N, Esmaeilzadeh A (2021). COVID-19: Virology, biology and novel laboratory diagnosis. J Gene Med.

[24] Ali H, Alahmad B, Al-Shammari AA, Alterki A, Hammad M, Cherian P (2021). Previous COVID-19 Infection and Antibody Levels After Vaccination. Front Public Health.

[25] Kwok SL, Cheng SM, Leung JN, Leung K, Lee CK, Peiris JM (2022). Waning antibody levels after COVID-19 vaccination with mRNA Comirnaty and inactivated CoronaVac vaccines in blood donors, Hong Kong, April 2020 to October 2021. Euro Surveill.

[26] Parczewski M, Kufel J, Aksak-Was B, Piwnik J, Chober D, Puzio T (2023). Artificial neural network based prediction of the lung tissue involvement as an independent in-hospital mortality and mechanical ventilation risk factor in COVID-19. J Med Virol.

[27] Kufel J, Bargiel K, Kozlik M, Czogalik L, Dudek P, Jaworski A (2022). Application of artificial intelligence in diagnosing COVID-19 disease symptoms on chest X-rays: A systematic review. Int J Med Sci.

[28] Rhodes S, Wilkinson J, Pearce N, Mueller W, Cherrie M, Stocking K (2022). Occupational differences in SARS-CoV-2 infection: analysis of the UK ONS COVID-19 infection survey. J Epidemiol Community Health.

[29] Mutambudzi M, Niedwiedz C, Macdonald EB, Leyland A, Mair F, Anderson J (2020). Occupation and risk of severe COVID-19: prospective cohort study of 120 075 UK Biobank participants. Occup Environ Med.

[30] Beale S, Hoskins S, Byrne T, Fong WLE, Fragaszy E, Geismar C (2023). Differential Risk of SARS-CoV-2 Infection by Occupation: Evidence from the Virus Watch prospective cohort study in England and Wales. J Occup Med Toxicol.

[31] Koźlik M, Kaźmierski M, Kaźmierski W, Lis P, Lis A, Łowicka W, Chamera M, Romanowska B, Kufel J, Cebula M, Jędrzejek M (2023). Quality of Life 6 Months after COVID-19 Hospitalisation: A Single-Centre Polish Registry. J Clin Med.

